# The influence of cone-beam computed tomography and endodontic practitioners’ proficiency level on diagnosis and treatment planning of root resorption

**DOI:** 10.4317/jced.62382

**Published:** 2025-05-01

**Authors:** Emel Olga Onay, Derin Bugu Yuzer, Eda Cakmak, Kamran Gulsahi

**Affiliations:** 1 Professor, Department of Endodontics, Faculty of Dentistry, Baskent University, Bahcelievler-Ankara, Turkey; 2 Research Fellow, Department of Endodontics, Faculty of Dentistry, Baskent University, Bahcelievler-Ankara, Turkey; 3 Assistant Professor, Department of Audiology, Faculty of Health Sciences, Baskent University, Bahcelievler-Ankara, Turkey

## Abstract

**Background:**

A precise diagnosis is essential for formulating an effective treatment plan for both internal and external resorptions. Cone-beam computed tomography (CBCT) offers a three-dimensional view of the maxillofacial area, capturing images from coronal, axial, and sagittal angles. This method overcomes the limitations of conventional intraoral radiography (IR), especially when it comes to detecting and identifying defects related to internal and external resorption. Thus, the aim of this study was to assess whether CBCT imaging affects the accuracy of diagnosing and planning treatment for internal and external resorption defects differently between endodontic residents (ERs) and specialists (ESs).

**Material and Methods:**

Thirty-five clinicians reviewed 3 internal and 3 external resorption cases using clinical histories and intraoral radiographs (IRs), then answered questions about their diagnosis and treatment decisions. One month later, they re-evaluated the cases with CBCT and answered similar questions. Data analyzed using Mc-Nemar chi-square test and Prevalence Adjusted Bias Adjusted Kappa statistic. The level of statistical significance was set off *p*< 0.05 in all data.

**Results:**

CBCT significantly improved diagnostic accuracy in 2 out of 6 cases (*p*< 0.001) and altered the treatment plan in 4 out of 6 cases (*p*< 0.05). There was no significant difference between ERs and ESs regarding diagnosis and treatment planning using the same imaging technique (*p*> 0.05).

**Conclusions:**

This study suggests that CBCT provides more detailed information compared to IR, with both imaging techniques allowing ERs and ESs to achieve similar diagnostic and treatment planning accuracy.

** Key words:**Clinical decision making, cone-beam computed tomography, dental radiography, diagnosis, root resorption.

## Introduction

Root resorption of permanent teeth refers to the loss of dental hard tissue (dentin and cementum) caused by odontoclastic activity (1). This condition is regarded as a pathological process that can potentially lead to tooth loss (2). Root resorptions are categorized into internal and external types based on their location. Internal resorption is further divided into inflammatory and replacement types, while external resorption is classified into several subtypes including surface, inflammatory, cervical, replacement, and transient apical breakdown, depending on their underlying causes (3).

Accurate diagnosis is crucial for developing an effective treatment strategy for internal and external resorptions. Currently, intraoral radiographs are commonly utilized due to their affordability and high image quality. Nevertheless, their diagnostic effectiveness is constrained by the complexity of anatomical structures and background interference (1,4).

Cone-beam computed tomography (CBCT) provides a 3D perspective of the maxillofacial region by capturing images in the coronal, axial, and sagittal planes (5). This technique addresses the shortcomings of traditional intraoral radiography (IR), particularly in the detection and identification of internal and external resorption defects (6).

Patel *et al*. (1) explored how CBCT scans compare to intraoral radiographs in diagnosing different types of resorption lesions. Their findings indicated that CBCT scans not only offered superior diagnostic accuracy but also enhanced the chances of proper management of these lesions. However, no research has yet examined how CBCT scans influence treatment planning among endodontic practitioners with varying levels of experience. Therefore, this study aimed to first determine whether preoperative CBCT scans could alter the diagnosis and treatment plans for internal and external resorption lesions when compared to IRs, and second, to evaluate the practitioners with different levels of experience in diagnosing and planning treatment for these resorption lesions.

## Material and Methods

Ethical approval for the study was issued by the Institutional Review Board at Baskent University (Project no: D-KA22/28). This study supported by Baskent University Research Fund.

-Case Selection and Radiographic Technique

A total of 6 teeth (3 exhibiting internal resorption and 3 with external root resorption) from 6 different patients were examined. All of these cases were treated by a specialist with more than 20 years of experience in endodontics.

**Figure 1:**
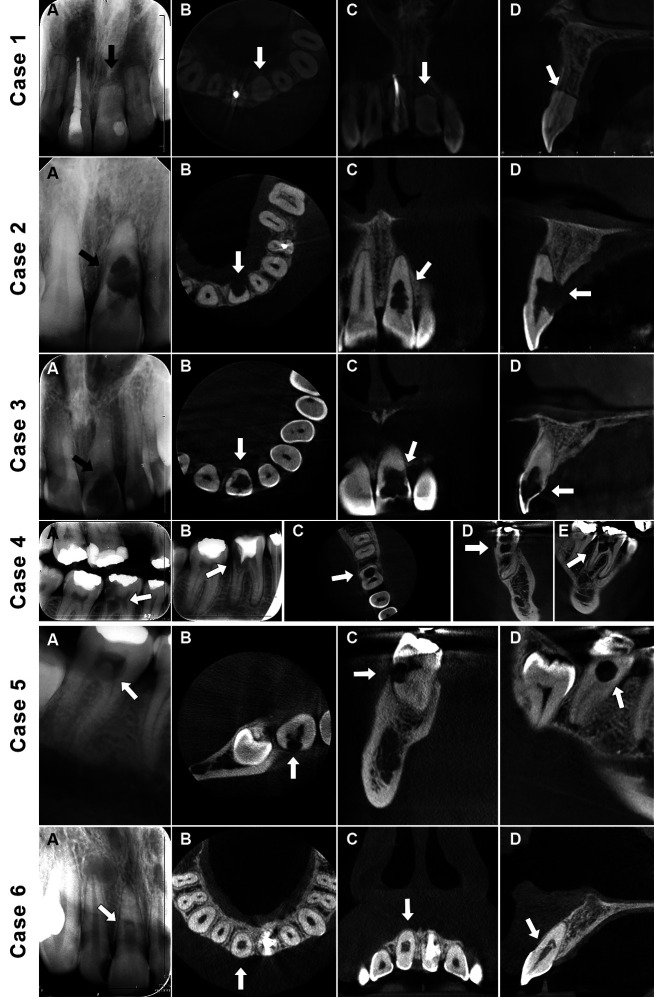
Case 1 (External apical root resorption): (A) A periapical radiograph of maxillary left central incisor. (B, C, D) CBCT reconstructed (axial, coronal, sagittal) views. Case 2 (External inflammatory resorption): (A) A periapical radiograph of maxillary left central incisor. (B, C, D) CBCT reconstructed (axial, coronal, sagittal) views. Case 3 (Internal inflammatory resorption): (A) A periapical radiograph of maxillary left central incisor. (B, C, D) CBCT reconstructed (axial, coronal, sagittal) views. Case 4 (Internal inflammatory resorption): (A) A bitewing radiograph of mandibular right first molar. (B) A periapical radiograph after treatment attempt by a general dentist. (C, D, E) CBCT reconstructed (axial, coronal, sagittal) views. Case 5 (External cervical resorption): (A) A periapical radiograph of mandibular right second molar. (B, C, D) CBCT reconstructed (axial, coronal, sagittal) views. Case 6 (Internal inflammatory resorption): (A) A periapical radiograph of maxillary right central incisor. (B, C, D) CBCT reconstructed (axial, coronal, sagittal) views. Arrows point to the teeth displaying resorption defects.

Digital IRs were captured using a Planmeca Dixi®3 sensor (Planmeca, Helsinki, Finland), set at 80 kVp, 8 mA, and 0.064 seconds (Fig. 1).

CBCT scans were acquired with a Morita 3D Accuitomo 170 (J Morita, Japan) using settings of 90 kVp, 5 mA, with a voxel size of 0.08 mm and a field of view measuring 40x40 mm. Image analysis was performed using i-Dixel software (version 2.2.1.6, Morita, Japan) on a medical monitor (Eizo Radiforce MX270W, Eizo Corporation, Japan) (Fig. 1).

-Study Participants

The eligibility requirements included being an endodontic resident (ER) or endodontic specialist (ES) with experience in using intraoral radiography and CBCT. Individuals who did not meet these criteria were excluded from participation. A group of male and female ERs (n=18) and ESs (n=17) who varied in age and clinical experience were selected ( Table 1). The sample size was determined using a prior estimation with a target power of 90% and a 95% confidence interval, leading to a calculated requirement of 32 participants. To account for potential dropouts and ensure the study’s integrity, a larger sample size was ultimately chosen.

Volunteers from the ER and ES groups completed 2 online surveys via Google Drive (Mountain View, CA, USA), with a one-month gap between them. Participants were informed clearly that joining the study was optional. They needed to respond to the first question of the survey to indicate their consent to participate before continuing with the survey. The initial survey included 6 clinical cases, each with detailed medical histories along with information on gender, age, and clinical signs and symptoms. Additionally, each case was accompanied by an IR. The survey included queries about the participants’ age, gender, years of endodontic practice, number of resorption cases identified both during and after endodontic training, and their general confidence in detecting resorption. Participants were required to select possible diagnoses (internal or external resorption), treatment methods (review, nonsurgical endodontic treatment, surgical endodontic treatment, combination of nonsurgical and surgical treatments, extraction), and their level of confidence (very confident, fairly confident, uncertain, fairly uncertain) regarding the chosen treatment for each case. The second survey (CBCT imaging) repeated the same clinical cases but incorporated limited FOV CBCT images and included questions similar to those in the initial survey.

As the study was retrospective, the expert panel established one final diagnosis and treatment approach for each case. This standard, considered the reference standard, was not revealed to the participants.

-Statistical Analysis 

Statistical analyses were conducted with Statistical Package for the Social Sciences (SPSS) version 25.0 (IBM Corp., Armonk, NY, USA). Qualitative data are summarized by numbers and percentages. Mc-Nemar chi-square test was used to evaluate two different imaging techniques for each case. In the binary diagnostic and treatment plan evaluation, the concordance analyses were tested with the PABAK (Prevalence Adjusted Bias Adjusted Kappa) statistic. The interpretation was based on the following reference: values of 0.80 or higher were considered to indicate almost perfect agreement; values between 0.61 and 0.80 signified substantial agreement; values from 0.41 to 0.60 reflected moderate agreement; values ranging from 0.21 to 0.40 represented regular agreement; and values of 0.20 or lower denoted slight agreement (7). The level of statistical significance was set off *p* < 0.05 in all data.

## Results

Demographic characteristics of participants, years of endodontic practice, number of resorption cases diagnosed during and after endodontic residency, and overall confidence level in diagnosing root resorption cases are summarized in Table 1.

-Comparison of the reference standard and radiographic diagnosis

The radiographic assessment aligned perfectly with the reference standard diagnosis in 35 out of 35 clinicians (100%) for case 1. For case 2, only 1 out of 35 clinicians (3%) matched the reference standard diagnosis. In case 3, 8 out of 35 clinicians (23%) were consistent with the reference standard diagnosis. For cases 4 and 5, 29 out of 35 clinicians (83%) agreed with the reference standard diagnosis. Finally, case 6 saw 30 out of 35 clinicians (86%) aligning with the reference standard diagnosis ( Table 2).

-Comparison of the reference standard and CBCT diagnosis

Out of 35 clinicians surveyed, 33 (94%) agreed with the reference standard diagnosis for case 1 based on CBCT, while 20 (57%) concurred for case 2, 26 (74%) for case 3, 28 (80%) for case 4, 30 (86%) for case 5, and 34 (97%) for case 6 ( Table 2).

-Comparison of IRs and CBCT scans against a reference standard diagnosis

The analysis of PABAK values indicates that the level of agreement between CBCT scans and IRs compared to the reference standard diagnosis was slight for cases 2 and 3, moderate for cases 4 and 5, and substantial for case 6. Since the radiographic diagnosis perfectly matched the reference standard diagnosis in all 35 clinicians surveyed (100%) for case 1, a PABAK value could not be calculated ( Table 2).

The CBCT group had a greater number of examiners aligning with the reference standard diagnosis compared to the IR group for cases 2, 3, 5, and 6. A significant difference between the two imaging techniques was observed for cases 2 and 3 (*p* < 0.001). However, no significant differences were noted between the two modalities for cases 5 and 6 (*p* > 0.05) ( Table 2).

Fewer examiners from the CBCT group agreed with the reference standard diagnosis in cases 1 and 4 compared to those in the IR group; however, no significant difference was observed between the two imaging methods for these cases (*p* > 0.05) ( Table 2).

-Changes in treatment plans based on a reference standard between IRs and CBCT scans

The analysis of PABAK values shows that the concordance between CBCT scans and IRs regarding the reference standard treatment plan choice was slight for cases 2 and 3, whereas regular for cases 1 and 5, and moderate for cases 4 and 6 ( Table 3).

A greater proportion of examiners using the CBCT method agreed with the reference standard treatment plan compared to those using IR for cases 2, 3, 5, and 6. Significant differences were observed between the two imaging techniques for cases 2 (*p* = 0.004), 3 (*p* = 0.035), and 5 (*p* = 0.022). However, no significant difference was found between the imaging methods for case 6 (*p* > 0.05) ( Table 3).

Fewer examiners in the CBCT group agreed with the reference standard treatment plan than those in the IR group for cases 1 and 4. In case 1, a significant difference was identified between the two imaging methods (*p* = 0.039). However, no significant difference was found for case 4 (*p* > 0.05) ( Table 3).

Comparison of ERs and ESs in diagnosing and planning treatment using the same imaging technique

There was no statistically significant difference between ERs and ESs in using the same imaging technique for establishing a reference diagnosis and developing a reference treatment plan for each case (*p* > 0.05) ( Table 4).

## Discussion

Studies have indicated that the use of CBCT enhances diagnostic accuracy in identifying resorption lesions. According to Lima *et al*. (8), CBCT outperformed digital periapical radiography in detecting both external and internal inflammatory root resorption following dental injuries. Furthermore, Madani *et al*. (9) highlighted CBCT’s effectiveness in diagnosing root resorptions. While current literature supports CBCT as a dependable method for spotting root resorption issues, IR also achieved a commendable level of precision in the present study. Kumar *et al*. (10) also found no significant difference in the accuracy of detecting defects between periapical radiographs and CBCT scans.

Since every case in this study was distinct, the extent and location of the resorption lesions differed between cases. In the present study, CBCT increased the diagnostic efficacy significantly in only 2 of the 6 cases. The lower-than-expected diagnostic efficiency of CBCT may be due to the particularly challenging nature of the cases and the fact that they were beyond the clinicians’ experience. Another possibility is that the results could be due to the observer’s confusion while interpreting the CBCT images (11).

While IRs are commonly used to diagnose internal and external resorption lesions, CBCT offers more detailed information, enhancing the likelihood of effective lesion management (1). In this study, CBCT significantly altered the treatment approach in 4 out of 6 cases. The results suggest that CBCT impacted the examiners’ recommendations for treatment. These results align with Rodriguez *et al*. (12), who found that CBCT imaging directly influences treatment decisions, especially among general practitioners, and with Ee *et al*. (13), who reported that CBCT imaging might influence treatment plans in approximately 62% of cases.

Whether endodontic training and clinical diagnostic skills influence diagnostic accuracy and proper treatment planning in resorption defects is still unknown. In this study, we examined whether CBCT imaging would have different effects on the accuracy of diagnosis and treatment planning of internal and external resorption defects among endodontic specialists (ESs) and endodontic residents (ERs). In this study, there was no statistically significant difference between ESs and ERs regarding the use of both imaging techniques for determining a reference standard diagnosis and developing a reference standard treatment plan for each case. In summary, the current findings confirm that both imaging techniques allowed ERs to diagnose and create treatment plans with the same level of accuracy as ESs. In line with our findings, Patel *et al*. (1) observed no difference in the diagnosis and treatment planning of resorption defects using CBCT imaging between postgraduate students and endodontists, although this finding was not the main goal of their study.

Routine use of CBCT imaging for diagnosing resorption defects is not recommended. However, it is advisable when resorption lesions are suspected based on regular IRs. Additionally, any exposure to ionizing radiation should adhere to the principle of “as low as reasonably achievable.” Therefore, the criteria for choosing and the parameters for each CBCT scan protocol should be carefully defined and adhere to the specific indications (8).

The main limitation of this study was the limited number of cases included. This small sample size was due to the rarity of the defects being studied, and it was based on data collected over 4 years by a single endodontist. Another constraint was the absence of standardized training for the observers, which may have affected consistency in interpreting the images. Due to challenges in recruiting all participants, it was not feasible to provide them with calibration or training prior to the analysis.

## Conclusions

The findings of this research suggest that while IRs are a valid diagnostic tool for internal and external resorption lesions, CBCT offers more detailed information. This improved clarity enhances the likelihood of effective lesion management. Additionally, the findings confirmed that both imaging techniques enabled ERs to diagnose and develop treatment plans with the same accuracy and efficiency as ESs.

## Figures and Tables

**Table 1:** Demographic characteristics of participants, years of endodontic practice, number of resorption cases diagnosed during and after endodontic residency, and overall confidence level in diagnosing root resorption cases.

	n (%)
Gender	
Female	23 (66)
Male	12 (34)
Years of experience	
0-5 years	18 (51)
6+ years	17 (49)
Total resorption cases diagnosed	
0-10 cases	11 (31)
11+ cases	24 (69)
Overall confidence level	
Highly confident	5 (14)
Moderately confident	18 (51)
Uncertain	8 (23)
Somewhat uncertain	2 (6)
Highly uncertain	2 (6)

**Table 2:** Comparison of intraoral radiographs and CBCT scans in relation to an established diagnostic standard.

n=35	Conclusive Diagnosis	IR n (%)	CBCT n (%)	PABAK	p
Case					
1	External apical root resorption	35 (100)	33 (94)	-	-
2	External inflammatory resorption	1 (3)	20 (57)	-0.086	<0.001
3	Internal inflammatory resorption	8 (23)	26 (74)	-0.143	<0.001
4	Internal inflammatory resorption	29 (83)	28 (80)	0.600	1.000
5	External cervical resorption	29 (83)	30 (86)	0.486	1.000
6	Internal inflammatory resorption	30 (86)	34 (97)	0.657	-

IR, Intraoral Radiography; CBCT, Cone-Beam Computed Tomography; PABAK, Prevalence Adjusted Bias Adjusted Kappa; p, significance level for Mc-Nemar test (*p*< 0.05).

**Table 3:** Differences in treatment plans according to a reference standard between intraoral radiographs and CBCT scans.

n=35	Conclusive Treatment	IR n (%)	CBCT n (%)	PABAK	p
Case					
1	Review	27 (77.1)	19 (54.3)	0.314	0.039
2	Extraction	4 (11.4)	16 (45.7)	0.086	0.004
3	Nonsurgical endodontic treatment	19 (54.3)	28 (80)	0.143	0.035
4	Nonsurgical endodontic treatment	28 (80)	26 (74.3)	0.429	0.754
5	Combination of nonsurgical and surgical endodontic treatments	4 (11.4)	11 (31.4)	0.257	0.022
6	Nonsurgical endodontic treatment	29 (82.9)	30 (85.7)	0.543	1.000

IR, Intraoral Radiography; CBCT, Cone-Beam Computed Tomography; PABAK, Prevalence Adjusted Bias Adjusted Kappa; p, significance level for Mc-Nemar test (*p*< 0.05).

**Table 4:** Diagnostic and treatment planning consistency between endodontic residents and specialists with the same imaging technique.

	IR (Diagnosis)	IR (Treatment Planning)
Case	p	p
1	-	0.289
2	-	0.375
3	0.312	0.344
4	0.375	0.125
5	0.125	0.375
6	0.375	0.687
	CBCT (Diagnosis)	CBCT (Treatment Planning)
Case	p	p
1	-	0.246
2	0.070	0.226
3	0.250	0.453
4	0.055	0.453
5	0.250	0.246
6	-	0.312

IR, Intraoral Radiography; CBCT, Cone-Beam Computed Tomography; p, significance level for Mc-Nemar test (*p*< 0.05).

## Data Availability

The datasets used and/or analyzed during the current study are available from the corresponding author.
